# Significance of physical factors on activities of daily living in patients with tetraplegia after spinal cord injury: a retrospective study

**DOI:** 10.1186/s13102-024-00928-z

**Published:** 2024-07-03

**Authors:** Kimin Yun, Jin-cheol Lim, Onyoo Kim

**Affiliations:** 1 Department of Physical Medicine and Rehabilitation, National Rehabilitation Center, 58, Samgaksan-ro, Gangbuk-gu, Seoul, 01022 Republic of Korea; 2 Department of Education Measurement and Evaluation, Sungkyunkwan University, Seoul, Korea

**Keywords:** Spinal cord injury, Tetraplegia, Rehabilitation, Functional ability, Physical factors, Activities of daily living, Korean spinal cord independence measure III

## Abstract

**Background:**

Tetraplegia is a debilitating sequela of spinal cord injury (SCI). However, comprehensive approaches for determining the influence of various factors on activities of daily living (ADL) in patients with tetraplegia are limited. Therefore, this study aimed to determine the influence of physical factors on ADL in patients with tetraplegia after adjusting for demographic, SCI-related, and cognitive factors.

**Methods:**

This retrospective cross-sectional study enrolled 201 patients with tetraplegia who underwent inpatient rehabilitation at the National Rehabilitation Center in South Korea between 2019 and 2021. Patients’ mean age was 50.5 years (standard deviation, 16.3), and 170 (84.6%) were men. The Korean Spinal Cord Independence Measure III (K-SCIM III) was used as the main outcome measure to assess patients’ ADL ability. Hierarchical multiple regression modeling was conducted with K-SCIM as the dependent variable to examine the level of functioning and relative influencing factors.

**Results:**

Upper-extremity motor score (UEMS), upper-extremity spasticity and sitting balance scores were significant predictors of self-care; lower-extremity motor score (LEMS), musculoskeletal pain of shoulder, and sitting balance were significant predictors of respiratory and sphincter management; UEMS, LEMS, and sitting balance score were significant predictors of mobility; and UEMS, LEMS, musculoskeletal pain of shoulder, and sitting balance scores were significant predictors of the K-SCIM III total score after adjustment for demographic, SCI-related, and cognitive factors.

**Conclusions:**

Physical factors had the greatest impact on all subscores and the K-SCIM III total score. Upper- and lower-extremity muscle strength and sitting balance significantly affected functional ability across all subscores.

## Background

Spinal cord injury (SCI) or damage to the spinal cord often results in severe functional impairments due to disruption of normal spinal cord anatomy [1]. Tetraplegia, which affects the arms, trunk, legs, and pelvic organs due to dysfunction or loss of motor and/or sensory function in the cervical segments of the spinal cord, is a particularly debilitating sequela of SCI [2]. Notably, SCI-related functional limitations can significantly affect patients’ quality of life (QOL) [3]. 

In rehabilitation medicine, assessing the ability to perform activities of daily living (ADL) is crucial for determining the degree of functional limitation and recovery [4]. The Spinal Cord Independence Measure (SCIM) is frequently used to assess daily activity performance in patients with SCI [5]. However, predicting the extent of recovery remains challenging because of the complex and multifaceted factors that influence functional ability in patients with tetraplegia. Therefore, further research is needed to identify factors that affect ADL and reliably predict recovery [6]. 

Functional outcomes of SCI are related to the following factors: SCI-related factors such as completeness [7–10], level of injury [10], and various other aspects including clinical characteristics such as age [10, 11], sex [11], BMI [11], nutritional status [12], comorbidities [9], and secondary complications [11]; physical factors such as spasticity [11, 13], contracture [14], upper-extremity motor score (UEMS) [15], and sitting balance [16, 17]; and psychosocial factors such as anxiety, depression [18], and insurance coverage [10, 11]. 

Despite efforts to identify the impact of the various factors that affect ADL on the lives of patients with tetraplegia, multifaceted studies that encompass these factors are limited. Therefore, our study aimed to investigate these influential factors in patients with tetraplegia by analyzing the Korean version of the Spinal Cord Independence Measure III’s (K-SCIM III) total score and subscores for self-care, respiratory and sphincter management, and mobility. Additionally, this study compared the relative impact of physical factors after adjusting for demographic, SCI-related, and cognitive factors.

## Methods

### Study design and participants

This retrospective cross-sectional study conformed to the Declaration of Helsinki guidelines and was approved by the institutional review board of the National Rehabilitation Center, Seoul (NRC-2022-04-028). It also adhered to the STROBE reporting guidelines. The requirement for informed consent was waived owing to the retrospective nature of the study design. The inclusion criteria of this study were patients with tetraplegia who received inpatient care between January 2019 and December 2021 at the National Rehabilitation Center in Seoul, South Korea and underwent detailed neurological assessments as well as K-SCIM according to the International Standards for Neurological Classification of SCI protocol. The exclusion criteria of this study were patients with missing values and those admitted for regular urodynamic study for a period of three days. This study enrolled 201 patients. Participants’ clinical data which included age, sex, duration and etiology of injury, neurological assessments, physical examinations, and the mini-mental state examination (MMSE) and K-SCIM III scores at the time of admission, were extracted from the medical records.

### Measurements

Etiology of injury was divided into traumatic and non-traumatic SCI, the latter category being in turn divided into six subcategories: tumor, myelitis, infection, spinal degeneration, arteriovenous malformation, and other causes. Duration of injury in non-traumatic injury was defined on the basis of the date of their initial hospital admission.

The neurological level of injury, American Spinal Injury Association impairment scale (AIS), UEMS, and lower-extremity motor score (LEMS) were determined on the basis of the International Standards for the Neurological Classification of SCI. The UEMS and LEMS are numerical summary scores of motor function for the upper and lower limbs, respectively. The maximum score is 25 for each extremity, totaling 50 for the upper and lower limbs, respectively [2]. 

Spasticity was assessed using the Modified Ashworth Scale (MAS). For convenience of statistical analysis, MAS grade 1 + was graded as point 2, and grades 2, 3, and 4, as points 3, 4, and 5, respectively. The upper-extremity spasticity score was calculated as the sum of the bilateral MAS scores for shoulder flexion, extension, and external and internal rotation; elbow flexion and extension; wrist flexion and extension; and finger flexion and extension, with a score ranging from 0 to 100. Lower-extremity spasticity score was defined as the sum of the bilateral MAS scores for hip flexion, extension, abduction, and adduction; knee flexion and extension; and ankle dorsiflexion and plantar flexion, with a score ranging from 0 to 80.

Sitting balance was assessed using the Sitting Balance scale [19]. It was scored as follows: normal, able to sit safely and securely for 2 min; good, able to sit for 2 min under supervision; fair, able to sit for 30 s; poor, able to sit for 10 s; or zero, unable to sit without support for 10 s.

The K-SCIM III was used to determine the level of functional ability post-SCI and was administered by occupational therapists specializing in SCI care [20]. The total K-SCIM III score ranges from 0 to 100, with higher scores reflecting higher levels of independence. The K-SCIM III has three subscores, namely self-care (subscore 1), respiratory and sphincter management (subscore 2), and mobility (subscore 3), ranging from 0 to 20, 0 to 40, and 0 to 40, respectively.

### Statistical analysis

All statistical analyses were performed using SPSS version 27.0. Significance was assessed using two-tailed tests with α-levels of 0.05. In our analyses, missing values were excluded using the listwise deletion method. Descriptive demographic data and clinical characteristics of the subjects were analyzed and presented as descriptive statistics, including means, standard deviations (SDs), and percentages. Hierarchical multiple regression modeling was conducted with K-SCIM as the dependent variable to examine the functioning level and relative influencing factors. A four-step process was followed for the modeling. In the first regression model, the demographic factors (age and sex) were included as independent variables. The second regression model included SCI-related factors (etiology, duration of injury, and AIS) as additional independent variables. The third regression model included cognitive factor (MMSE) as an additional independent variable. Measures of physical factors (UEMS, LEMS, upper- and lower-extremity spasticity, limitation of shoulder and hip ROMs, shoulder musculoskeletal pain, and sitting balance) were added to the final regression model. The F-value was calculated to verify the validity of the hierarchical regression analysis. To verify whether the addition of a new factor significantly improved the predictive power of the regression model, the change in the coefficient of determination (R²) was examined for each additional factor. The K-SCIM III total score and each subscore were analyzed.

## Results

### Demographic and clinical characteristics of the participants

Demographic and baseline clinical characteristics of the participants are listed in Table 1.

**Table 1:** Demographics and baseline clinical characteristics of participants (*N* = 201)

Factor	Variable	Category	Mean ($\text{S}\text{D}$)	*N* (%)
Demographic factors	Age (years)		50.5 (16.3)	201 (100.0)
	Sex	Male		170 (84.6)
		Female		31 (15.4)
SCI-related factors	AIS	A		51 (25.4)
		B		35 (17.4)
		C		26 (12.9)
		D		89 (44.3)
	NLI	C-2		9 (4.5)
		C-3		23 (11.4)
		C-4		76 (37.8)
		C-5		65 (32.3)
		C-6		15 (7.5)
		C-7		6 (3.0)
		C-8		3 (1.5)
		T-1		4 (2.0)
	Duration of Injury (year)	$<$ 1 year		123 (61.2)
		$\ge$ 1 year		78 (38.8)
	Etiology of Injury	Traumatic		167 (83.1)
		Nontraumatic		34 (16.9)
Cognitive factors	MMSE		28.1 (2.8)	201 (100.0)
Physical factors	UEMS		24.2 (12.7)	201 (100.0)
	LEMS		17.3 (17.5)	201 (100.0)
	Upper-extremity spasticity		8.1 (9.5)	201 (100.0)
	Lower-extremity spasticity		12.4 (12.1)	201 (100.0)
	Limitation of shoulder ROM	Yes		120 (59.7)
		No		81 (40.3)
	Limitation of hip ROM	Yes		43 (21.4)
		No		158 (78.6)
	Musculoskeletal pain of shoulder	Yes		83 (41.3)
		No		118 (58.7)
	Musculoskeletal pain of hip	Yes		0 (0.0)
		No		0 (0.0)
	Sitting balance	Good		35 (17.4)
		Fair		43 (21.4)
		Poor		54 (26.9)
		Zero		69 (34.3)
K-SCIM-III	Self-care sub score		3.8 (4.9)	201 (100.0)
	Respiration and sphincter management sub score		19.4 (9.9)	201 (100.0)
	Mobility sub score		6.8 (8.5)	201 (100.0)
	Total SCIM score		30.0 (20.6)	201 (100.0)

*Abbreviations* AIS, American Spinal Injury Association Impairment Scale; NLI, Neurological Level of Injury; MMSE, Mini-mental State Examination; UEMS, Upper-Extremity Motor Score; LEMS, Lower-Extremity Motor Score; ROM, Range of Motion; K-SCIM, Korean Spinal Cord Independence Measure

The mean age was 50.5 years (SD, 16.3), and 170 (84.6%) participants were men. The numbers of participants with AIS A, B, C, and D were 51 (25.4%), 35 (17.4%), 26 (12.9%), and 89 (44.4%), respectively. The most common neurological levels of injury were C4 and C5 in 76 (37.8%) and 65 (32.3%) participants, respectively. A total of 123 (61.2%) participants had an injury duration of < 1 year. The number of participants with traumatic SCI was 167 (83.1%), while 34 (16.9%) had non-traumatic SCI, with spinal degeneration accounting for 13 (6.4%) as the major cause. The mean K-MMSE was 28.1 (SD, 2.8). The mean UEMS and LEMS were 24.2 (SD, 12.7) and 17.3 (SD, 17.5), respectively, and mean upper- and lower-extremity spasticity scores were 8.1 (SD, 9.5) and 12.4 (SD, 12.1), respectively. A total of 120 (59.7%) and 43 (21.4%) participants had limited shoulder and hip ROMs, respectively, while 83 (41.3%) had shoulder musculoskeletal pain. A total of 35 (17.4%), 43 (21.4%), 54 (26.9%), and 69 (34.3%) patients had good, fair, poor, and zero sitting balance scores, respectively. The subscores 1, 2, 3, and the K-SCIM III total scores were 3.8 (SD, 4.9), 19.4 (SD, 9.9), 6.8 (SD 8.5), and 30.0 (SD 20.6), respectively.

### Predictors of the K-SCIM III as measured by hierarchical regression analysis

The results of the hierarchical multiple regression analyses of the factors that influenced subscores 1, 2, 3, and the total K-SCIM III score are listed in Tables 2, 3 and 4, and 5, respectively. Model 1 examined the impact of demographic factors on functional ability. In models 2, 3, and 4, new variables were introduced while adjusting for the previously entered variables, and the impact of each variable on functional ability and the R² and F-values for the variables within each model were analyzed.

**Table 2:** Hierarchical regression analyses of predictors of K-SCIM III subscore 1

Model (Factor)	Variable	B	$\beta$	t	*p*	*R* [2] ($\varDelta$*R* [2])
Model 1 Demographic factors	(Constant)	9.04	—	6.93	< 0.001	0.091 (0.091)
	Age (years)	-0.05	-0.16	-2.30	**0.023**	
	Sex	-3.41	-0.25	-3.74	**< 0.001**	
Model 2 SCI-related factor	(Constant)	14.32	—	10.61	< 0.001	0.302 (0.211)
	Age (years)	-0.07	-0.25	-3.93	**< 0.001**	
	Sex	-1.94	-0.14	-2.23	**0.027**	
	Etiology of Injury	-4.23	-0.33	-4.96	**< 0.001**	
	AIS	-3.03	-0.31	-4.86	**< 0.001**	
	Duration of Injury (year)	-0.55	-0.06	-0.90	0.372	
Model 3 Cognitive factors	(Constant)	2.35	—	0.67	0.502	0.348 (0.046)
	Age (years)	-0.06	-0.19	-3.03	**0.003**	
	Sex	-2.02	-0.15	-2.39	**0.018**	
	Etiology of Injury	-4.07	-0.31	-4.93	**< 0.001**	
	AIS	-2.64	-0.27	-4.32	**< 0.001**	
	Duration of Injury (year)	-0.46	-0.05	-0.76	0.448	
	MMSE	0.38	0.22	3.69	**< 0.001**	
Model 4 Physical factors	(Constant)	-0.26	—	-0.09	0.931	0.585 (0.237)
	Age (years)	-0.03	-0.11	-1.82	0.070	
	Sex	-1.23	-0.09	-1.70	0.091	
	Etiology of Injury	-2.18	-0.17	-3.08	**0.002**	
	AIS	0.38	0.04	0.43	0.671	
	Duration of Injury (year)	-0.74	-0.07	-1.42	0.158	
	MMSE	0.15	0.09	1.63	0.104	
	UEMS	0.17	0.44	6.37	**< 0.001**	
	LEMS	0.02	0.08	0.74	0.460	
	Upper-extremity spasticity	-0.08	-0.15	-2.32	**0.021**	
	Lower-extremity spasticity	0.04	0.09	1.55	0.123	
	Limitation of shoulder ROM	0.24	0.02	0.39	0.698	
	Limitation of hip ROM	-0.55	-0.05	-0.92	0.359	
	Musculoskeletal pain of shoulder	-0.62	-0.06	-1.10	0.274	
	Sitting balance	1.66	0.17	2.52	**0.013**	

Model1: $\varDelta$F (2, 198) = 9.86 (*p* < .001); Model2: $\varDelta$F (3, 195) = 16.69 (*p* < .001); Model3: $\varDelta$F (1, 194) = 13.60 (*p* < .001); Model4: $\varDelta$F (8, 186) = 13.27 (*p* < .001)

**Dummy variables**: Sex (ref = female); Etiology of injury (ref = Nontraumatic); AIS (ref = C and D); Duration of Injury (ref = over than 1 year); Limitation of shoulder ROM (ref = No); Limitation of hip ROM (ref = No); Musculoskeletal pain of shoulder (ref = No); Sitting balance (ref = Poor and Zero)

*Abbreviations* AIS, American Spinal Injury Association Impairment Scale; MMSE, Mini-mental State Examination; UEMS, Upper-Extremity Motor Score; LEMS, Lower-Extremity Motor Score; ROM, Range of Motion; K-SCIM III, Korean Spinal Cord Independence Measure

**Table 3:** Hierarchical regression analyses of predictors of the K-SCIM III subscore 2

Model (Factor)	Variable	B	$\beta$	t	*p*	*R* 2 ($\varDelta$*R* 2)
Model 1 Demographic factors	(Constant)	17.83	—	6.49	< 0.001	0.016 (0.016)
	Age (years)	0.06	0.10	1.49	0.138	
	Sex	-1.95	-0.07	-1.02	0.310	
Model 2 SCI-related factor	(Constant)	30.89	—	12.32	< 0.001	0.413 (0.397)
	Age (years)	-0.04	-0.06	-1.12	0.263	
	Sex	-0.53	-0.02	-0.33	0.745	
	Etiology of Injury	-5.79	-0.22	-3.66	**< 0.001**	
	AIS	-11.28	-0.57	-9.75	**< 0.001**	
	Duration of Injury (year)	0.89	0.04	0.77	0.441	
Model 3 Cognitive factors	(Constant)	10.38	—	1.59	0.114	0.445 (0.033)
	Age (years)	-0.01	-0.02	-0.29	0.776	
	Sex	-0.67	-0.02	-0.42	0.674	
	Etiology of Injury	-5.52	-0.21	-3.58	**< 0.001**	
	AIS	-10.62	-0.53	-9.29	**< 0.001**	
	Duration of Injury (year)	1.06	0.05	0.94	0.347	
	MMSE	0.66	0.19	3.38	**0.001**	
Model 4 Physical factors	(Constant)	6.13	—	1.10	0.274	0.637 (0.192)
	Age (years)	-0.02	-0.03	-0.58	0.559	
	Sex	-0.02	0.00	-0.02	0.986	
	Etiology of Injury	-3.35	-0.13	-2.49	**0.014**	
	AIS	1.01	0.05	0.59	0.555	
	Duration of Injury (year)	0.12	0.01	0.12	0.905	
	MMSE	0.39	0.11	2.27	**0.024**	
	UEMS	-0.01	-0.01	-0.16	0.870	
	LEMS	0.36	0.64	6.49	**< 0.001**	
	Upper extremity spasticity	-0.02	-0.02	-0.32	0.751	
	Lower extremity spasticity	-0.04	-0.05	-0.88	0.380	
	Limitation of shoulder ROM	-0.27	-0.01	-0.23	0.818	
	Limitation of hip ROM	0.26	0.01	0.23	0.820	
	Musculoskeletal pain of shoulder	-2.59	-0.13	-2.42	**0.016**	
	Sitting balance	3.71	0.18	2.97	**0.003**	

Model1: $\varDelta$F (2, 198) = 1.59 (*p* < .207); Model2: $\varDelta$F (3, 195) = 43.92 (*p* < .001); Model3: $\varDelta$F (1, 194) = 11.45 (*p* < .001); Model4: $\varDelta$F (8, 186) = 12.29 (*p* < .001)

**Dummy variables**: Sex (ref = female); Etiology of injury (ref = Nontraumatic); AIS (ref = C and D); Duration of Injury (ref = over than 1 year); Limitation of shoulder ROM (ref = No); Limitation of hip ROM (ref = No); Musculoskeletal pain of shoulder (ref = No); Sitting balance (ref = Poor and Zero)

*Abbreviations* AIS, American Spinal Injury Association Impairment Scale; MMSE, Mini-mental State Examination; UEMS, Upper-Extremity Motor Score; LEMS, Lower-Extremity Motor Score; ROM, Range of Motion; K-SCIM III, Korean Spinal Cord Independence Measure

**Table 4:** Hierarchical regression analyses of predictors of the K-SCIM III subscore 3

Model (Factor)	Variable	B	$\beta$	t	*p*	*R*2 ($\varDelta$*R*2)
Model 1 Demographic factor	(Constant)	8.30	—	3.44	0.001	0.003 (0.003)
	Age (years)	-0.01	-0.01	-0.18	0.858	
	Sex	-1.28	-0.05	-0.76	0.448	
Model 2 SCI-related factors	(Constant)	18.00	—	7.28	< 0.001	0.249 (0.246)
	Age (years)	-0.07	-0.13	-1.97	**0.050**	
	Sex	0.25	0.01	0.16	0.876	
	Etiology of Injury	-4.98	-0.22	-3.20	**0.002**	
	AIS	-7.53	-0.44	-6.61	**< 0.001**	
	Duration of Injury (year)	-0.90	-0.05	-0.80	0.426	
Model 3 Cognitive factors	(Constant)	-2.06	—	-0.32	0.750	0.290 (0.041)
	Age (years)	-0.04	-0.07	-1.14	0.257	
	Sex	0.12	0.00	0.07	0.941	
	Etiology of Injury	-4.72	-0.21	-3.10	**0.002**	
	AIS	-6.89	-0.40	-6.11	**< 0.001**	
	Duration of Injury (year)	-0.74	-0.04	-0.67	0.504	
	MMSE	0.64	0.21	3.36	**0.001**	
Model 4 Physical factors	(Constant)	-8.31	—	-1.54	0.125	0.554 (0.264)
	Age (years)	-0.04	-0.07	-1.17	0.242	
	Sex	1.18	0.05	0.89	0.377	
	Etiology of Injury	-1.74	-0.08	-1.34	0.181	
	AIS	4.61	0.27	2.80	**0.006**	
	Duration of Injury (year)	-1.94	-0.11	-2.04	**0.042**	
	MMSE	0.28	0.09	1.69	0.093	
	UEMS	0.14	0.21	2.92	**0.004**	
	LEMS	0.31	0.63	5.70	**< 0.001**	
	Upper extremity spasticity	-0.08	-0.09	-1.28	0.203	
	Lower extremity spasticity	0.01	0.01	0.21	0.836	
	Limitation of shoulder ROM	0.44	0.03	0.38	0.701	
	Limitation of hip ROM	-1.18	-0.06	-1.09	0.278	
	Musculoskeletal pain of shoulder	-1.29	-0.07	-1.25	0.213	
	Sitting balance	3.25	0.19	2.69	**0.008**	

Model1: $\varDelta$F (2, 198) = 0.31 (*p* < .735); Model2: $\varDelta$F (3, 195) = 21.26 (*p* < .001); Model3: $\varDelta$F (1, 194) = 11.27 (*p* < .001); Model4: $\varDelta$F (8, 186) = 13.76 (*p* < .001)

**Dummy variables**: Sex (ref = female); Etiology of injury (ref = Nontraumatic); AIS (ref = C and D); Duration of Injury (ref = over than 1 year); Limitation of shoulder ROM (ref = No); Limitation of hip ROM (ref = No); Musculoskeletal pain of shoulder (ref = No); Sitting balance (ref = Poor and Zero)

Abbreviations: AIS, American Spinal Injury Association Impairment Scale; MMSE, Mini-mental State Examination; UEMS, Upper-Extremity Motor Score; LEMS, Lower-Extremity Motor Score; ROM, Range of Motion; K-SCIM III, Korean Spinal Cord Independence Measure

**Table 5:** Hierarchical regression analyses of predictors of the K-SCIM III total score

Model (Factor)	Variable	B	$\beta$	t	*p*	*R*2 ($\varDelta$*R*2)
Model 1 Demographic factors	(Constant)	35.17	—	6.10	< 0.001	0.014 (0.014)
	Age (years)	0.01	0.01	0.12	0.908	
	Sex	-6.64	-0.12	-1.65	0.101	
Model 2 SCI-related factors	(Constant)	63.20	—	11.74	< 0.001	0.383 (0.369)
	Age (years)	-0.18	-0.14	-2.41	**0.017**	
	Sex	-2.22	-0.04	-0.64	0.524	
	Etiology of Injury	-15.00	-0.27	-4.41	**< 0.001**	
	AIS	-21.83	-0.52	-8.79	**< 0.001**	
	Duration of Injury (year)	-0.57	-0.01	-0.23	0.818	
Model 3 Cognitive Factor	(Constant)	10.68	—	0.77	0.442	0.432 (0.049)
	Age (years)	-0.11	-0.08	-1.43	0.155	
	Sex	-2.57	-0.05	-0.77	0.443	
	Etiology of Injury	-14.31	-0.26	-4.37	**< 0.001**	
	AIS	-20.15	-0.48	-8.31	**< 0.001**	
	Duration of Injury (year)	-0.14	0.00	-0.06	0.954	
	MMSE	1.69	0.23	4.09	**< 0.001**	
Model 4 Physical factors	(Constant)	-2.44	—	-0.22	0.823	0.685 (0.254)
	Age (years)	-0.09	-0.07	-1.37	0.171	
	Sex	-0.08	0.00	-0.03	0.976	
	Etiology of Injury	-7.27	-0.13	-2.78	**0.006**	
	AIS	6.00	0.14	1.80	0.073	
	Duration of Injury (year)	-2.56	-0.06	-1.34	0.183	
	MMSE	0.81	0.11	2.44	**0.016**	
	UEMS	0.30	0.18	3.08	**0.002**	
	LEMS	0.69	0.59	6.35	**< 0.001**	
	Upper extremity spasticity	-0.18	-0.08	-1.42	0.156	
	Lower extremity spasticity	0.01	0.00	0.07	0.943	
	Limitation of shoulder ROM	0.41	0.01	0.18	0.859	
	Limitation of hip ROM	-1.47	-0.03	-0.67	0.503	
	Musculoskeletal pain of shoulder	-4.49	-0.11	-2.16	**0.032**	
	Sitting balance	8.63	0.20	3.53	**< 0.001**	

Model1: $\varDelta$F (2, 198) = 1.36 (*p* < .258); Model2: $\varDelta$F (3, 195) = 38.89 (*p* < .001); Model3: $\varDelta$F (1, 194) = 16.71 (*p* < .001); Model4: $\varDelta$F (8, 186) = 18.73 (*p* < .001)

**Dummy variables**: Sex (ref = female); Etiology of injury (ref = Nontraumatic); AIS (ref = C and D); Duration of Injury (ref = over than 1 year); Limitation of shoulder ROM (ref = No); Limitation of hip ROM (ref = No); Musculoskeletal pain of shoulder (ref = No); Sitting balance (ref = Poor and Zero)

*Abbreviations* AIS, American Spinal Injury Association Impairment Scale; MMSE, Mini-mental State Examination; UEMS, Upper-Extremity Motor Score; LEMS, Lower-Extremity Motor Score; ROM, Range of Motion; K-SCIM III, Korean Spinal Cord Independence Measure

Regarding the factors affecting subscore 1 of the K-SCIM III, model 1, which used demographic factors as control variables, was statistically significant (F = 9.86, *P* < .001, ${R}^{2\hspace{0.17em}}$= 0.091). Model 2 was statistically significant (F = 16.69, *P* < .001, ${R}^{2}\hspace{0.17em}$= 0.302) after adjusting for demographic factors, and model 3 was statistically significant (F = 13.60, *P* < .001, ${R}^{2}\hspace{0.17em}$= 0.348) after adjusting for demographic and SCI-related factors, with MMSE ($\beta \hspace{0.17em}$= 0.22, *P* < .001) as a significant predictor. Model 4 was statistically significant as well (F = 13.27, *P* < .001, ${R}^{2}\hspace{0.17em}$= 0.585) after adjusting for all previous factors. In model 4, the UEMS ($\beta \hspace{0.17em}$= 0.44, *P* < .001), upper extremity spasticity ($\beta \hspace{0.17em}$= − 0.15, *P* = .021), and sitting balance scale ($\beta \hspace{0.17em}$= 0.17, *P* = .013) scores were significant predictors.

Regarding the factors affecting subscore 2 of the K-SCIM III, model 1, which used demographic factors as control variables, was not statistically significant (F = 1.59, *P* = .207, ${R}^{2}\hspace{0.17em}$= 0.016). Model 2 was statistically significant (F = 43.92, *P* < .001, ${R}^{2}\hspace{0.17em}$= 0.413) after adjusting for demographic factors. Model 3 was statistically significant (F = 11.45, *P* = .001, ${R}^{2}\hspace{0.17em}$= 0.445) after adjusting for demographic and SCI-related factors, with MMSE ($\beta \hspace{0.17em}$= 0.19, *P* = .001) as a significant predictor, and model 4 was statistically significant (F = 12.29, *P* < .001, ${R}^{2}\hspace{0.17em}$= 0.637) after adjusting for all previous factors. In Model 4, LEMS ($\beta \hspace{0.17em}$= 0.64, *P* < .001), musculoskeletal pain of shoulder ($\beta \hspace{0.17em}$= − 0.13, *P*=.016), and sitting balance ($\beta \hspace{0.17em}$= 0.18, *P* = .003) were significant predictors.

Regarding the factors affecting subscore 3 of the K-SCIM III, Model 1, which used demographic factors as control variables, was not statistically significant (F = 0.31, *P* = .207, ${R}^{2}\hspace{0.17em}$= 0.016). Model 2 was statistically significant (F = 21.26, *P* < .001, ${R}^{2}\hspace{0.17em}$= 0.249) after adjusting for demographic factors. Model 3 was statistically significant (F = 11.27, *P* = .001, ${R}^{2}$ = 0.290) after adjusting for demographic and SCI-related factors, with MMSE ($\beta \hspace{0.17em}$= 0.21, *P* = .001) as a significant predictor, and model 4 was statistically significant (F = 13.76, *P* < .001, ${R}^{2}\hspace{0.17em}$= 0.554) after adjusting for all previous factors. In Model 4, UEMS ($\beta \hspace{0.17em}$= 0.21, *P* = .004), LEMS ($\beta$ = 0.63, *P* < .001) and sitting balance score ($\beta \hspace{0.17em}$= 0.19, *P* = .008) were significant predictors.

Regarding the factors affecting the total K-SCIM III score, Model 1, which used demographic factors as control variables, was not statistically significant (F = 1.36, *P* = .258, ${R}^{2}\hspace{0.17em}$= 0.014). Model 2 was statistically significant (F = 38.89, *P* < .001, ${R}^{2}$= 0.383) after adjusting for demographic factors. Model 3 was statistically significant (F = 16.71, *P* < .001, ${R}^{2}\hspace{0.17em}$= 0.432) after adjusting for demographic and SCI-related factors, with MMSE ($\beta \hspace{0.17em}$= 0.23, *P* < .001) as a significant predictor. Model 4 was statistically significant (F = 18.73, *P* < .001, ${R}^{2}$ = 0.685) after adjusting for all previous factors. In Model 4, UEMS ($\beta \hspace{0.17em}$= 0.18, *P* = .002), LEMS ($\beta \hspace{0.17em}$= 0.59, *P* < .001), musculoskeletal pain of shoulder ($\beta \hspace{0.17em}$= − 0.11, *P* = .032) and sitting balance ($\beta \hspace{0.17em}$= 0.20, *P* < .001) scores were significant predictors.

## Discussion

Our study investigated the multiple factors that influenced ADL in patients with tetraplegia by analyzing the K-SCIM III total score and subscores. Using hierarchical multiple regression analysis, we found that physical factors had the greatest impact on ADL after adjusting for demographic, SCI-related, and cognitive factors.

Our findings also suggested that upper-extremity strength and spasticity as well as sitting balance can significantly affect self-care in patients with tetraplegia. Patients with SCI usually present with self-care deficits and depend on caregivers for basic ADL [21]. Upper-extremity strength is strongly correlated with self-care, particularly grooming [15]. Spasticity can interfere with hand or limb control and can significantly impact ADL [22]. Spasms, a sign of spasticity, are reportedly associated with self-care [13]. Additionally, sitting balance [23] significantly impacts self-care. Patients with tetraplegia perform most ADLs in a seated position; hence, they rate trunk stability as a priority for improving independence [24]. Our study revealed that sitting balance and upper-extremity factors such as muscle strength are important in self-care functions in patients with tetraplegia. Therefore, rehabilitation strategies should focus on improving trunk stability to enhance self-care functions.

After adjusting for demographic, SCI-related, and cognitive factors, our study found that muscle strength and sitting balance were significant correlations of mobility. The mobility component of the K-SCIM III comprises two subscales; “room and toilet” and “indoors and outdoors on even surface.” [25] The subscales include all types of mobility, whether using a wheelchair or walking aids. Enhanced mobility predicted improved self-perceived health, higher life satisfaction, and greater community participation [26]. Dynamic activities such as propelling a wheelchair up or down ramps often require sitting balance control, and unsupported sitting balance is important for efficient transfer performance [16]. Additionally, muscle strength is a key determinant of mobility score [27, 28]. Therefore, rehabilitation therapy should focus on muscle strength, including the UEMS and LEMS, and sitting balance.

South Korea is experiencing a trend toward rapid population aging. The age at which traumatic SCI occurs has gradually increased from 32.4 years in 1990 to 40.1 years in 2000 and 47.1 years in 2010. This is especially evident in the group aged 40–49 years, with the highest value observed in the group aged > 70 years [29]. Additionally, most older adults lose the ability to live independently because of cognitive disabilities [30]. The importance of cognitive factors as significant correlates of functional disability in patients with SCI is often overlooked. Our study found that cognitive factors were significantly correlated with all subscores and the total K-SCIM III score. Additionally, it is important to note that patients with traumatic brain injury were not included in our study.

A distinctive aspect of our approach was the adjustment for demographic, SCI-related, and cognitive factors when assessing the influence of physical factors on ADL. Many studies have found that age [10, 11], sex [11], and completeness [7–10] and level of injury [10] influence ADL. As these factors can confound the results, we analyzed the impact of physical factors on ADL. Consequently, our results emphasize the significance of physical factors in determining ADL in patients with tetraplegia, even after adjusting for potential confounding factors. This suggests that physical factors influence functional outcomes and that targeted interventions are needed to address these factors in rehabilitation strategies.

Despite valuable insights, our study has certain limitations owing to its retrospective design. First, the study data were limited by the quality and completeness of the medical records, which can be subject to errors and omissions. Second, although this study demonstrated associations between variables, establishing causality was difficult because of the cross-sectional study design. Third, this study was based on the medical records of patients at the National Rehabilitation Center in Seoul, South Korea; therefore, the findings may not be generalizable to the entire population. Fourth, previous studies have suggested the influence of nutritional status, psychological factors, medical complications, and funder classification on functional abilities [10, 12, 18, 31]. However, in this study, these unmeasured variables were potential confounding biases that limited the interpretability of our findings. Lastly, the lack of subgroup analysis results represents a limitation of this study. The absence of such analyses prevented a deeper understanding of mobility differences between wheelchair users and ambulators; consequently, future research should prioritize comparisons between various subgroups to inform the development of more effective rehabilitation strategies.

## Conclusions

This study revealed that after adjusting for demographic, SCI-related, and cognitive factors, physical factors—especially muscle strength and sitting balance—had the greatest impact on all K-SCIM III subscores and the total score. Demographic and SCI-related factors are unmodifiable; hence, rehabilitation strategies should focus on these physical factors to optimize functional outcomes and enhance the overall QOL of patients with tetraplegia.

## Data Availability

The datasets used and/or analysed during the current study are available from the corresponding author on reasonable request.

## Abbreviations

SCI: Spinal cord injury
QOL: Quality of life (QOL)
ADL: Activities of daily living
SCIM: Spinal Cord Independence Measure
K-SCIM III: Korean version of the Spinal Cord Independence Measure III
UEMS: Upper-extremity motor score
LEMS: Lower-extremity motor score
AIS: Association impairment scale
MAS: Modified Ashworth Scale
MMSE: Mini-mental state examination
